# Responses of Phosphate Transporter Gene and Alkaline Phosphatase in *Thalassiosira pseudonana* to Phosphine

**DOI:** 10.1371/journal.pone.0059770

**Published:** 2013-03-27

**Authors:** Mei Fu, Xiuxian Song, Zhiming Yu, Yun Liu

**Affiliations:** 1 Key Laboratory of Marine Ecology and Environmental Sciences, Institute of Oceanology, Chinese Academy of Sciences, Qingdao, China; 2 University of Chinese Academy of Sciences, Beijing, China; Louisiana State University and A & M College, United States of America

## Abstract

Phosphine, which is released continuously from sediment, can affect the eco-physiological strategies and molecular responses of phytoplankton. To examine the effects of phosphine on phosphorus uptake and utilization in *Thalassiosira pseudonana*, we examined the transcriptional level of the phosphate transporter gene (*Tp*PHO) and the activity of alkaline phosphatase (AKP) in relation to supplement of various concentrations of phosphine. *Tp*PHO expression was markedly promoted by phosphine in both the phosphate-deficient and phosphate-4 µM culture. However, high phosphine concentrations can inhibit *Tp*PHO transcription in the declining growth phase. AKP activity was also higher in the phosphine treatment groups than that of the control. It increased with increasing phosphine concentration in the range of 0 to 0.056 µM but was inhibited by higher levels of phosphine. These responses revealed that phosphine can affect phosphate uptake and utilization in *T. pseudonana*. This result was consistent with the effect of phosphine on algal growth, while *Tp*PHO expression and AKP were even more sensitive to phosphine than algal growth. This work provides a basic understanding for further research about how phosphine affects phytoplankton.

## Introduction

Phosphorus (P) is essential for the growth of planktonic algae and plays a significant role in cell physiology and biochemistry in the marine environment. Increasing evidence indicates that P is frequently a limiting nutrient for phytoplankton, and its absence can inhibit primary productivity [1]–[3]. Previously, P was commonly assumed existing in the environment as fully oxidized phosphate. Along with the development of analytical technology, reduced inorganic forms of P, including phosphite, hypophosphite, phosphide and phosphine (PH_3_), have been frequently detected in the natural environment [4]. Therefore, it is likely that organisms with the ability to utilize reduced P as alternative sources of P may be at an ecological advantage under P limiting conditions. Recent biochemical evidence suggests that some organisms can utilize phosphite and hypophosphite as alternative sources of P [4]. However, whether phosphine can serve as an alternative source of P under P limiting conditions is still not known.

Phosphine, even as a trace component, is a ubiquitous constituent of the hydrosphere [5]–[10]. It exists in two forms: free gaseous phosphine and matrix-bound phosphine (MBP) [10]–[14]. MBP, normally presented in soils, sediments, sludge, and other condensed media, can hydrolyze in biological aquatic media to form free phosphine gas. Phosphine is a reduced compound that can be easily oxidized to other forms of P. Therefore, phosphine should not be neglected in the marine environment considering its potential ecological roles to marine organisms. Besides, phosphine is commonly used as a fumigant to control insects that affect stored products throughout the world [15]–[19]. It is toxic to animals such as mites [15], insects [15]–[17], and mammals [18]. The effect of phosphine on animals has been examined in various studies [18], [19], but only a few focused primarily on the effects of phosphine on phytoplankton (e.g. *Microcystis aeruginosa* [20]). Niu et al. [20] reported that phosphine stimulated the growth of *M*. *aeruginosa* and that the release of phosphine may play an important role in outbreaks of algal blooms in eutrophic waters. However, little is known about what the fate of phosphine is and how it affects marine phytoplankton.

Changes in expression of phosphate transporter genes and activity of alkaline phosphatase (AKP) can be useful for investigating the effects of phosphine on microalgae and how it is utilized by microalgae. Dong et al. [21] reported a strong relationship between phosphate uptake and the expression of phosphate transporter genes. Other researchers have studied phosphorus uptake by cells [22], and the first step is believed to be transport of phosphorus across the cell membrane by a membrane-associated transporter. Willsky et al. [23] proposed that phosphorus uptake is maintained by two distinct systems: the Pit (phosphate inorganic transport) system and the Pst (phosphate specific transport) system. Pit is a low-affinity, high-velocity system and Pst is a high-affinity, low-velocity system. A high-affinity phosphate transporter gene encoding a previously described Pst system was identified in several plankton species, such as *Anabaena* sp. PCC7120 [24] and *Tetraselmis chui* [25]. Furthermore, its expression was regulated by the phosphate concentration in the medium. Low phosphate concentrations also can induce AKP activity. This enzyme hydrolyzes various forms of organic P and releases P, making it available for direct uptake [26]. Like phosphate transporter genes, AKP is another bioreporter of P metabolism [24]. Vrba et al. [27] reported that algae might adapt to phosphorus depletion by increasing AKP activity.

*Thalassiosira pseudonana* was used to investigate the mRNA expression of a high-affinity phosphate transporter gene and the activity of AKP in response to phosphine. This species is a common red tide algae and its full genome has been sequenced making this species an attractive model organism in genetic studies [28]–[30]. The nucleotide sequence of high-affinity phosphate transporter gene in *T. pseudonana*, *Tp*PHO, has been deposited in the GenBank database under accession number XM002292288.1. In this study, we investigated the effects of phosphine on P uptake and utilization by *T. pseudonana* by assessing the responses of the phosphate transporter gene and AKP to various levels of phosphine. Our results provides basic understanding of how phosphine affects phytoplankton and will benefit further research to explore the fate of phosphine in the oceanic biogeochemical cycle of phosphorus.

## Materials and Methods

### Algae and Culture Conditions

*T*. *pseudonana* was provided by Xiamen University, Fujian, China. The unialgal stock and experimental cultures were maintained at 20±1°C under a light:dark cycle of 12∶12 h, with 80 µmol photon m^−2^s^−1^ provided by four cool-white ﬂuorescent light bulbs. The stock culture was grown in L_1_-enriched seawater medium [31] and maintained in the exponential growth phase by inoculating cells into fresh L_1_ medium every 5 d. *T. pseudonana* was cultured at low P concentration (4 µM) for at least five generations before the experiments were started. Glassware was washed with ∼10% v/v HCl to remove possible contaminants, rinsed with distilled water, and then autoclaved at 121 kPa for 30 min.

Cultures at mid-exponential phase were inoculated into 2.5 L of sterilized seawater in 3L sealable culture ﬂasks. The initial cell density for all experiments was on average 5.45×10^5^ cells/ml, and they were grown under identical conditions as the stock culture. Nutrients, except phosphate (PO_4_^3−^), were added to the cultures. All experimental cultures were inoculated for 24 h to ensure that the original algal cells exhausted the P in the medium before the addition of P to the medium, in between each ﬂask was agitated gently every 12 h.

### Experimental Design

To test the effects of phosphine on the transcriptional level of *Tp*PHO, nine treatments with different initial P concentrations were prepared (Table 1). According to the concentration of phosphine in the marine environment [8] and the preliminary experiment, we chose 0.022 and 0.22 µmol/l (represent low-PH_3_ and high-PH_3_ respectively) for the experiment. Samples were collected at different growth stages (1, 2, 4, and 6 d) for analysis of *Tp*PHO transcription. The timing of phosphine introduction also is described in Table 1.

**Table 1:** Control and P conditions for *Tp*PHO gene expression experiment.

Treatment	Description
Control (no-PH_3_ treatment in P-deficient culture)	The initial added concentrations of phosphate and phosphine were both 0 µM
0.022 µM PH_3_ (low-PH_3_ treatment in P-deficient culture)^a^	The initial added concentration of PO_4_^3−^ was 0 µM, and 0.022 µM PH_3_ was introduced into the medium every day
0.022 µM PO_4_^3−^ (low- PO_4_^3-^ treatment in P-deficient culture)^a^	The initial added concentration of PO_4_^3−^ was 0 µM, and 0.022 µM PO_4_^3−^ was introduced into the medium every day
0.22 µM PH_3_ (high-PH_3_ treatment in P-deficient culture)^a^	The initial added concentration of PO_4_^3−^ was 0 µM, and 0.22 µM PH_3_ was introduced into the medium every day
0.22 µM PO_4_^3−^ (high-PO_4_^3-^ treatment in P-deficient culture)^a^	The initial added concentration of PO_4_^3−^ was 0 µM, and 0.22 µM PO_4_^3−^ was introduced into the medium every day
2 µM PO_4_^3−^ +0.022 µM PH_3_ (low-PH_3_ treatment in phosphate-2 µMculture)^b^	The initial added concentration of PO_4_^3−^ was 2 µM, and 0.022 µM PH_3_ was introduced into the medium every day
4 µM PO_4_^3−^ +0.022 µM PH_3_ (low-PH_3_ treatment in phosphate-4 µMculture)^b^	The initial added concentration of PO_4_^3−^ was 4 µM, and 0.022 µM PH_3_ was introduced into the medium every day
4 µM PO_4_^3−^ +0 µM PH_3_ (no-PH_3_ treatment in phosphate-4 µM culture) b	The initial concentration of PO_4_^3−^ was 4 µM, and no PH_3_ was introduced
4 µM PO_4_^3−^ +0.22 µM PH_3_ (high-PH_3_ treatment in phosphate-4 µMculture)^b^	The initial concentration of PO_4_^3−^ was 4 µM, and 0.22 µM PH_3_ was introduced into the medium every day

a The concentrations of phosphine or phosphate were added for the first time at 24 h after inoculation and then added after sampling each morning.

b Phosphate was added at 24 h after inoculation, and phosphine was introduced first at the same time. Thereafter, phosphine was introduced after sampling each morning.

To examine the effects of phosphine on AKP activity, initial phosphine concentrations were 0 µM, 0.022 µM, 0.056 µM, and 0.22 µM in cultures, respectively. No phosphate (0 µM) was added to the treatments. Nitrate, silicate and other nutrients were the same as in L_1_ medium. Phosphine was introduced into the phosphate-depleted culture at 24 h after inoculation. Cultures were sampled at 3, 6, 24 and 48 h after the addition of phosphine.

All treatments were carried out in triplicate.

### Growth Parameters

During the course of the *Tp*PHO experiments, cell densities of the algae were determined every day. After illumination for 2 h each day, ∼10 ml of algal suspension were sampled from the culture and fixed with Lugol’s solution for determination of cell densities. Cell densities were determined using a light microscope (Nikon Eclipse 50i, Tokyo, Japan) at a magnification of ×400 and were used to calculate the specific growth rate (*µ*) of the algae with the equation: *µ* = (ln*N_2_*− ln*N_1_*)/(*t_2_*− *t_1_*), where *N_1_* and *N_2_* represent cell numbers at *t_1_* and *t_2_*, respectively, and *t* is the incubation time.

### Extraction of Total RNA and First-strand cDNA Synthesis

The samples from the *Tp*PHO experimental cultures at 1, 2, 4, and 6 d were centrifuged at 3500 rev/min (2512×g) for 5 min at 20°C. Approximately 10^8^ cells were harvested and stored in liquid nitrogen. Cells were disrupted by grinding in lysis buffer containing β-mercaptoethanol. The total RNA was extracted using the RNAprep pure Plant Kit (TIANGEN Biotech, Beijing, China) following the manufacturer’s instructions. The isolated RNA was resuspended in 30 µl RNase-free water. RNA concentration was determined using a spectrophotometer (Thermo Scientific, NanoDrop 1000, Waltham, MA, USA) and the purity was estimated by the 260/280 nm absorption ratio (all ratios ranged from 1.8 to 2.1). RNA integrity was verified by agarose gel electrophoresis. RNA of appropriate quality was obtained for all samples.

DNase I-treated total RNA (1 µg) from each of the different samples was reverse transcribed into cDNA using an oligo(dT)_18_ primer (Sangon Biotech, Shanghai, China) and PrimeScript reverse transcriptase (TaKaRa, Kyoto, Japan) according to the manufacturer’s instructions.

### Real-time Quantitative RT-PCR

First-strand cDNA was synthesized as described above. Real-time quantitative RT-PCR, based on the SYBR Green method was performed using SYBR Premix Ex Taq™ (TaKaRa) with a real-time thermal cycler system (Eppendorf, Hamburg, Germany). The PCR cycle program included an initial denaturation at 95°C for 30 s for 1 cycle, followed by 40 cycles at 95°C for 5 s, 60°C for 20 s, and 72°C for 30 s. A melting curve analysis was conducted to verify formation of a single unique product and the absence of potential primer dimerization. Triplicate qRT-PCR assays were performed for each sample [32].

To standardize the amount of total RNA present in each reaction, the housekeeping gene (internal reference gene) 18S rRNA was co-amplified and used as the standard. Primers were chosen using the Primer Premier 5 program to have a melting temperature of 58–60.4°C. Primers (Table 2) were synthesized at Sangon Biotech. PCR products were sequenced (Sangon Biotech) to verify the validity of these primers.

**Table 2:** List of primers and probes used for real-time RT-PCR.

Gene name	Sequence (5′→3′)	Tm(°C)	Amplicon length
*Tp*18SrRNAf	TCTTAGTTGGTGGAGTGATTTGTC	58.0	140 bp
*Tp*18SrRNAr	CGCCATCTTCCTTCATCTTGTA	59.6	
*Tp*PHOf	TCTTCTATGCGGATGCCTGAG	60.4	157 bp
*Tp*PHOr	GCCGTGGCACATTGTTCTG	60.2	

Amplification efficiency of all RT-PCR reactions was analyzed through serial dilutions of cDNA originating from the control culture. Transcriptional levels of the target genes were calculated using the 2^–ΔΔ*C*^ *_T_* method [33] according to the equation ΔΔ*C_T_* = (*C_T, Target_*-*C_T, 18s rRNA_*)_sample_-(*C_T, Target_*-*C_T, 18s rRNA_*)_control_. The *C_T_* values of three runs of the same sample were averaged before carrying out the ΔΔ*C_T_* calculation. The relative abundance of *Tp*PHO transcripts was 2^–ΔΔ*C*^ *_T_* fold relative to the control (fold is the unit for *Tp*PHO transcriptional level).

### AKP Activity

Samples (taken from cultures for each treatment at 3, 6, 24, and 48 h) used to measure cellular AKP activity were collected onto 25 mm diameter Whatman GF/F glass fiber filter membranes (pre-combusted at 450°C for 5 h) and stored at –20°C. Blank filters were prepared similarly. Cells were disrupted by grinding in Tris-HCl buffer (pH  = 7.8) in an external ice-bath, and AKP activity then was determined using an AKP assay kit (Nanjing Jiancheng Bioengineering Institute, Nanjing, China) following the manufacturer’s instructions.

### Statistical Analysis

The software SPSS 17.0 for Windows was used for statistical analysis. One-way analysis of variance (ANOVA) with Tukey’s multiple comparison test was applied to test differences in cell densities, specific growth rates, *Tp*PHO transcription, and AKP activity of different phosphine treatments. A significance level of 0.05 was used in the analysis.

## Results

### Algal Growth Under Different P-conditions

**Figure 1:**
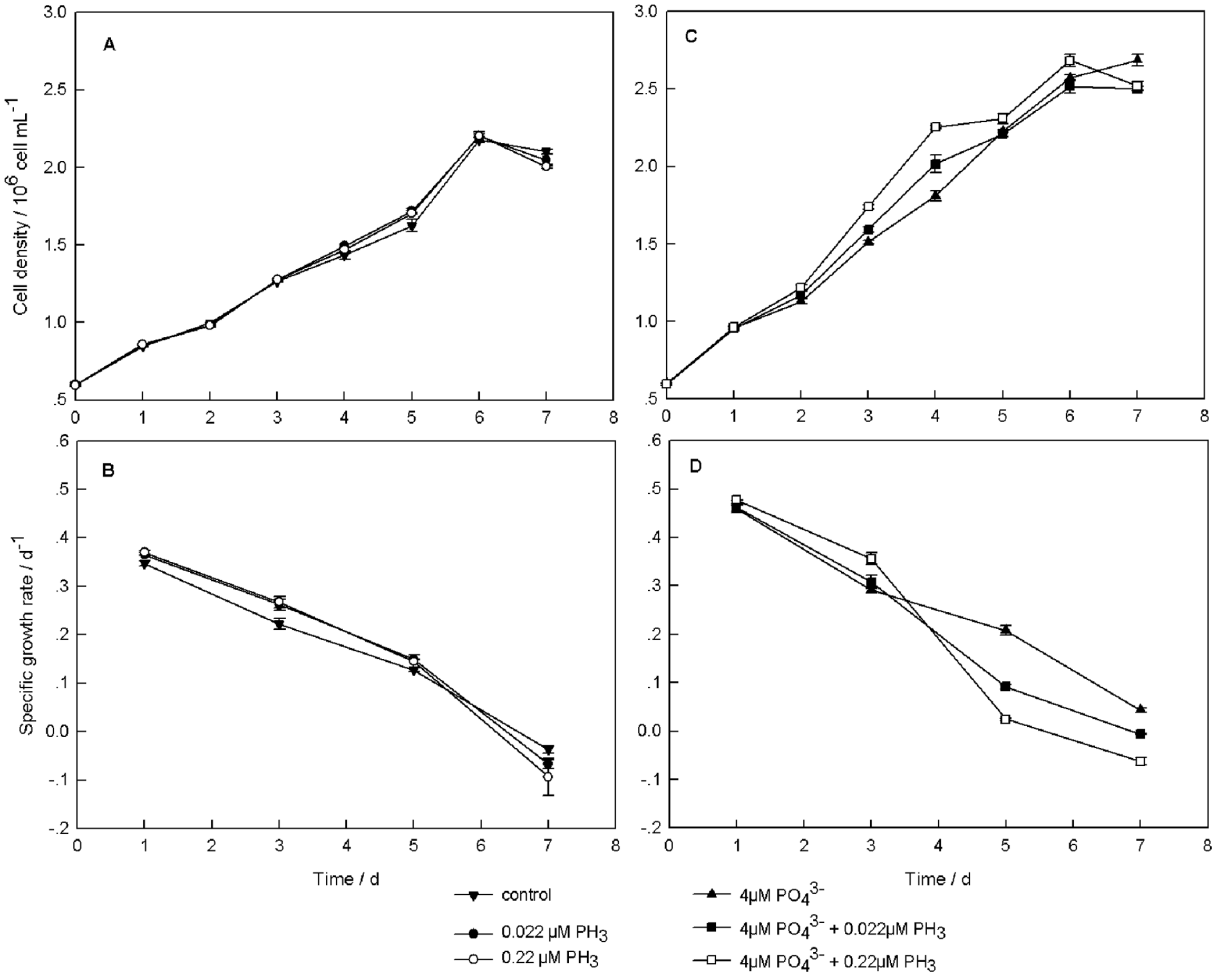
Algal growth parameters of *T*. *pseudonana* with different phosphine levels. (A) Cell density under the phosphate-deficient condition. (B) Growth rate under the phosphate-deficient condition. (C) Cell density under the phosphate-4 µM condition. (D) Growth rate under the phosphate-4 µM condition. The error bars represent standard deviations about the mean. For data points without an error bar, the error bar is smaller than the symbol.

When *T. pseudonana* was cultured in phosphate-deficient medium (PO_4_^3−^  = 0 µM), the cell density increased during the first 6 days of the experiment. On day 6, the cell density of control (0 µM PH_3_), low-PH_3_ treatment (0.022 µM PH_3_), and high-PH_3_ treatment (0.22 µM PH_3_) reached 2.17×10^6^, 2.20×10^6^, 2.20×10^6^ cells mL^−1^, respectively, but the culture reached the stationary stage on day 7. In this condition, different concentrations of phosphine had no significant effect on cell density (P>0.05) (Figure 1A). In the phosphate-deficient medium, specific growth rate (*μ*) in the high-PH_3_ treatment was higher than in the low-PH_3_ treatment, and both of the phosphine treatments had a higher *µ* than the control during the early culture period (day 1 and day 3, Figure 1B). The highest *μ* was 0.370 d^−1^ in the high-PH_3_ treatment on day 1.When incubation time was prolonged, specific growth rates of all treatments decreased. On day 5, there were no significant differences among the three phosphate-deficient treatments with different concentrations of phosphine (P>0.05). The specific growth rates of the three phosphate-deficient treatments were lower than 0 d^−1^ on day 7. The cell mortality rate in the high-PH_3_ treatment was higher than that in the low-PH_3_ treatment, and both of the phosphine treatments had a higher mortality rate than the control. The mortality rate was 0.094 d^−1^ for the high-PH_3_ treatment, 0.068 d^−1^ for the low-PH_3_ treatment, and 0.038 d^−1^ for the control.

In the phosphate-4 µM medium (PO_4_^3−^  = 4 µM), *T. pseudonana* also grew during the first 6 d and reached the stationary stage on day 7. The maximum cell density was 2.69×10^6^ cells mL^−1^ in the high-PH_3_ treatment on day 6 and the no-PH_3_ treatment on day 7. Different cell densities were found in different treatments beginning on day 3, and the most pronounced difference occurred on day 4. The cell density of the high-PH_3_ treatment was higher than that of the low-PH_3_ treatment and no-PH_3_ treatment from days 2 to 6. The low-PH_3_ treatment had higher cell density than the no-PH_3_ treatment (Figure 1C). Figure 1D shows specific growth rates of the phosphine treatments in the phosphate-4 µM culture. The variation among the three different phosphine treatments was similar to the variation observed in the phosphate-deficient medium on days 1 and 3. The highest *µ* was 0.477 d^−1^ in the high-PH_3_ treatment on day 1. On day 5, *µ* of the no-PH_3_ treatment was higher than that of the low-PH_3_ treatment; and *µ* of the high-PH_3_ treatment was lowest among these treatments. On day 7, *µ* of the no-PH_3_ treatment was higher than 0 d^−1^. However, the mortality rate was 0.063 d^−1^ in the high-PH_3_ treatment and 0.006 d^−1^ in the low-PH_3_ treatment. Compared to the phosphate-deficient cultures, the phosphate-4 µM cultures had higher specific growth rates.

### Amplification Efficiencies of *Tp*PHO and 18SrRNA

**Figure 2:**
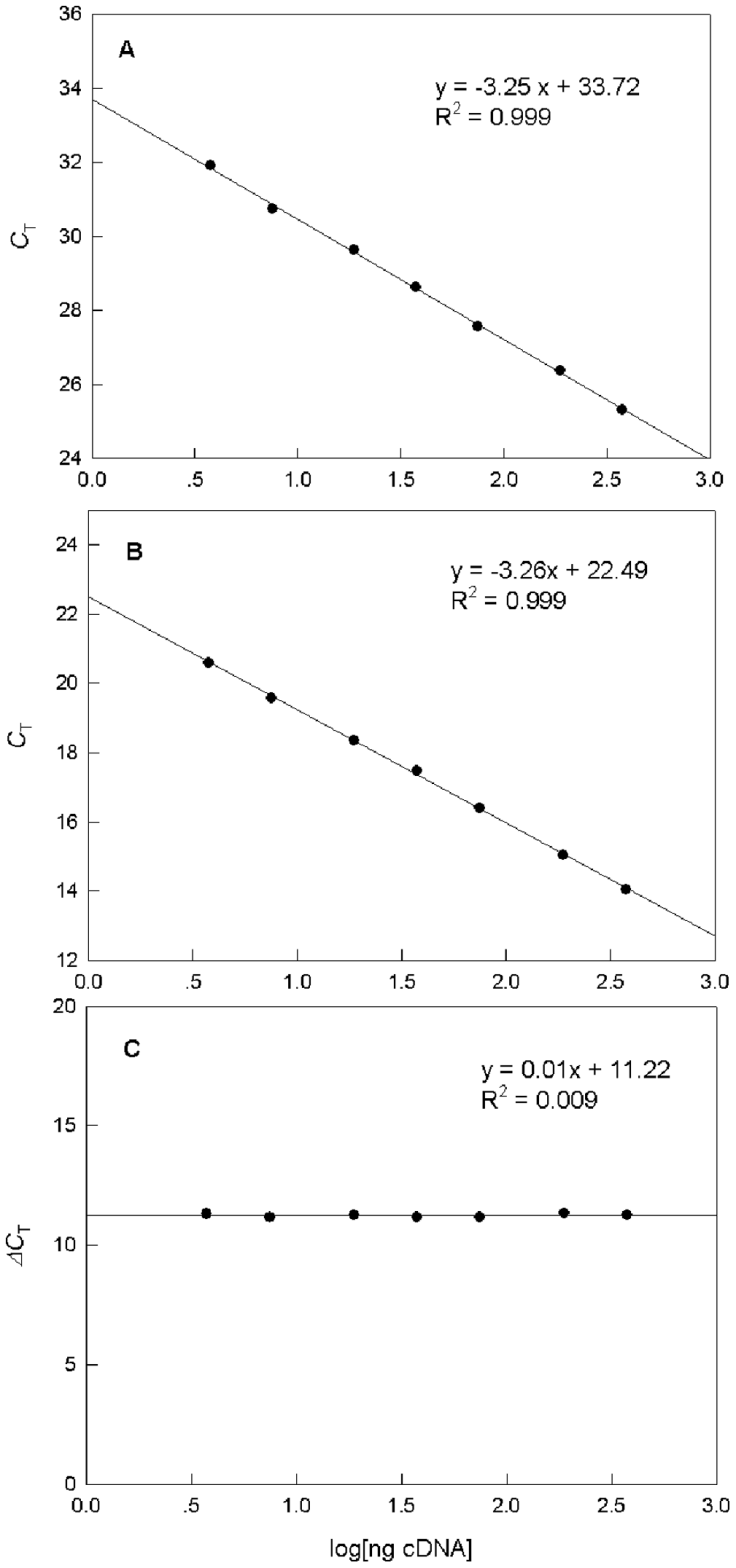
Amplification efficiencies of *Tp*PHO and 18SrRNA. Real-time quantitative RT-PCR standard curves of (A) *Tp*PHO and (B) 18S rRNA. (C) Δ*C*_T._ Curves were generated by plotting the logarithm of various cDNA concentrations (3 to 400 ng) versus *C*_T_ or Δ*C*_T_ values.

To validate the 2^–ΔΔ*C*^ *_T_* method, all reactions were ensured to have the same amplification efficiencies. To assess the homogeneity of amplification efficiencies of the target and reference genes, variation of Δ*C_T_* in serial dilutions of template cDNA was evaluated. Figure 2 shows the standard curves of log cDNA diluted over a 200-fold range. The threshold cycles (*C_T_*) of *Tp*PHO and 18SrRNA were both linearly correlated with log cDNA dilution. Slopes of the standard curves were –3.25 for *Tp*PHO (Figure 2A) and –3.26 for 18SrRNA (Figure 2B). Both were close to the theoretical value of –3.32. A plot of Δ*C*_T_ (*C*_T, *Tp*PHO_ – *C*_T, 18S_) versus log cDNA dilution (Figure 2C) revealed that the value of the slope was 0.01, which is close to zero. Therefore, the amplification efficiencies of the two genes were equal, and the 2^–ΔΔ*C*^ *_T_* method could be used to analyze the data [33].

### Effect of Phosphine on Transcriptional Level of *Tp*PHO

**Figure 3:**
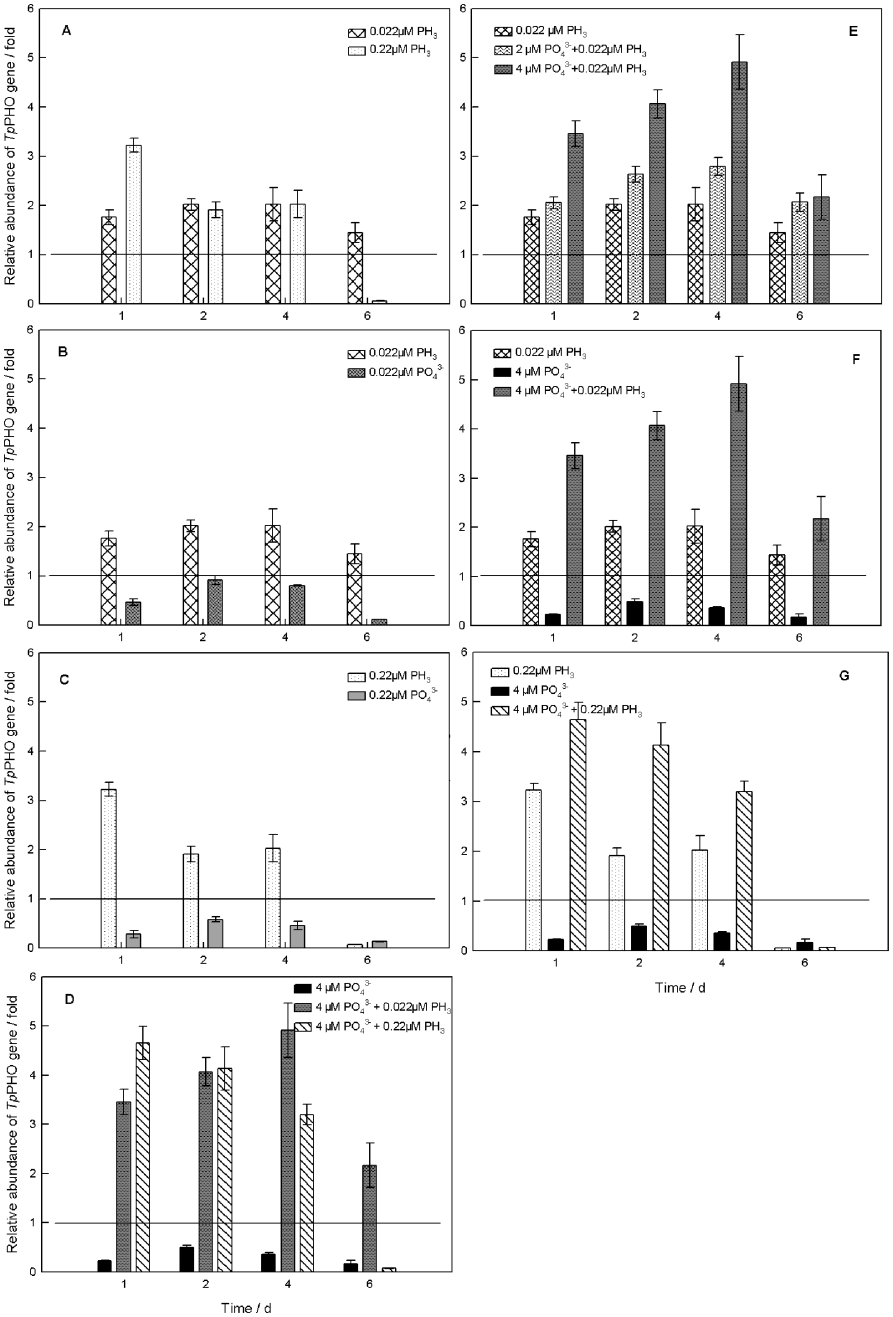
Effects of phosphine on transcriptional level of *Tp*PHO in *T*. *pseudonana*. The horizontal lines show that the relative abundance of *Tp*PHO in the control (reference group) was 1 fold. (A) Under the phosphate-deficient condition, different concentrations of phosphine were introduced. (B) Under the phosphate-deficient condition, the same low concentrations (0.022 µM) of phosphine and phosphate were added. (C) The same high concentrations (0.22 µM) of phosphine and phosphate were added, respectively, under the phosphate-deficient condition. (D) Different concentrations of phosphine were added under the phosphate-4 µM condition. (E) The low concentration (0.022 µM) of phosphine was introduced into cultures with different initial concentrations of phosphate. (F) and (G) Phosphine and phosphate have a synergistic effect. For all treatments, the first sampling occurred at 24 h (day 1) after the first addition of phosphate and/or phosphine, which was 48 h after inoculation. The error bars represent standard deviations.

In the phosphate-deficient culture, the *Tp*PHO transcriptional level increased with increased concentration of phosphine on the first day. The *Tp*PHO transcriptional level in the low-PH_3_ (0.022 µM PH_3_) treatment was 1.760 fold relative to the control and the transcriptional level in the high-PH_3_ (0.22 µM PH_3_) treatment was 3.226 fold. The relative abundance of *Tp*PHO transcripts in the low-PH_3_ treatment increased on days 2 and 4 and decreased on day 6, but it still was higher than the abundance in the control (>1 fold). In contrast, the *Tp*PHO transcriptional level decreased with culture age in the high-PH_3_ treatment. The lowest level of *Tp*PHO transcription among all samples tested was observed in this treatment on day 6 (0.064 fold) (Figure 3A).

Two treatments of phosphate in the same concentrations as phosphine (0.022 µM, 0.22 µM), also were tested under phosphate-deficient conditions. The *Tp*PHO transcriptional level of these two PO_4_^3−^ treatments was lower than that of the control (<1 fold), and much lower than those of the phosphine treatments (Figure 3B and 3C).

The temporal change in *Tp*PHO transcriptional level in the phosphate-4 µM cultures was similar to that observed in the phosphate-deficient culture (Figure 3D). After 1 day, the *Tp*PHO transcriptional level increased with the concentration of phosphine. In the phosphate-4µM culture, the *Tp*PHO transcriptional level in the low-PH_3_ (4 µM PO_4_^3−^ +0.022 µM PH_3_) treatment was 3.455 fold. It was lower than the level in the high-PH_3_ (4 µM PO_4_^3−^ +0.22 µM PH_3_) treatment (4.652 fold) but much higher than the level in the no-PH_3_ (4 µM PO_4_^3−^ +0 µM PH_3_) treatment (0.228 fold). After 2 days, the *Tp*PHO transcriptional level in both the low-PH_3_ and no-PH_3_ treatments increased slightly, and the low-PH_3_ treatment reached the highest level (4.913 fold) on day 4. As in the phosphate-deficient culture, the *Tp*PHO transcriptional level in the low-PH_3_ treatment (2.17 fold) was higher than that in the control. In contrast, the level in the high-PH_3_ treatment was significantly lower than that in the control after day 6. The transcriptional level in the no-PH_3_ treatment was always lower than that in the phosphine treatments and in the control.

Figure 3E shows the effect of phosphine on the *Tp*PHO transcriptional level in the treatments with different initial concentrations of phosphate. For the low-PH_3_ treatments with different concentrations of phosphate (0 µM PO_4_^3−^ +0.022 µM PH_3_, 2 µM PO_4_^3−^ +0.022 µM PH_3_, 4 µM PO_4_^3−^ +0.022 µM PH_3_), the *Tp*PHO transcriptional level increased with increased phosphate concentration. The *Tp*PHO levels of all three treatments increased over the first 4 days of the experiment and then decreased by day 6. The differences in transcript levels among the three treatments on day 6 were not significant (P>0.05).

Figure 3F illustrates a synergistic effect between phosphine and phosphate on transcription of *Tp*PHO. On day 1, the relative abundances of *Tp*PHO in the low-PH_3_ treatment (0 µM PO_4_^3−^ +0.022 µM PH_3_) and phosphate-4 µM treatment (4 µM PO_4_^3−^ +0 µM PH_3_) were 1.760 fold and 0.228 fold, respectively. However, the *Tp*PHO transcriptional level of the low-PH_3_ treatment in the phosphate-4 µM culture (4 µM PO_4_^3−^ +0.022 µM PH_3_) dramatically increased to 4.070 fold. Moreover, throughout the experiment, the TpPHO gene expression of the 4 µM PO_4_^3−^ +0.022 µM PH_3_ treatment was higher than the sum of the other two treatments. The synergistic effect could be illustrated by the high- PH_3_ treatment (0 µM PO_4_^3−^ +0.22 µM PH_3_), phosphate-4 µM treatment (4 µM PO_4_^3−^ +0 µM PH_3_), and phosphate-4 µM culture (4 µM PO_4_^3−^ +0.22 µM PH_3_) (Figure 3G).

### Effect of Phosphine on AKP Activity

**Figure 4:**
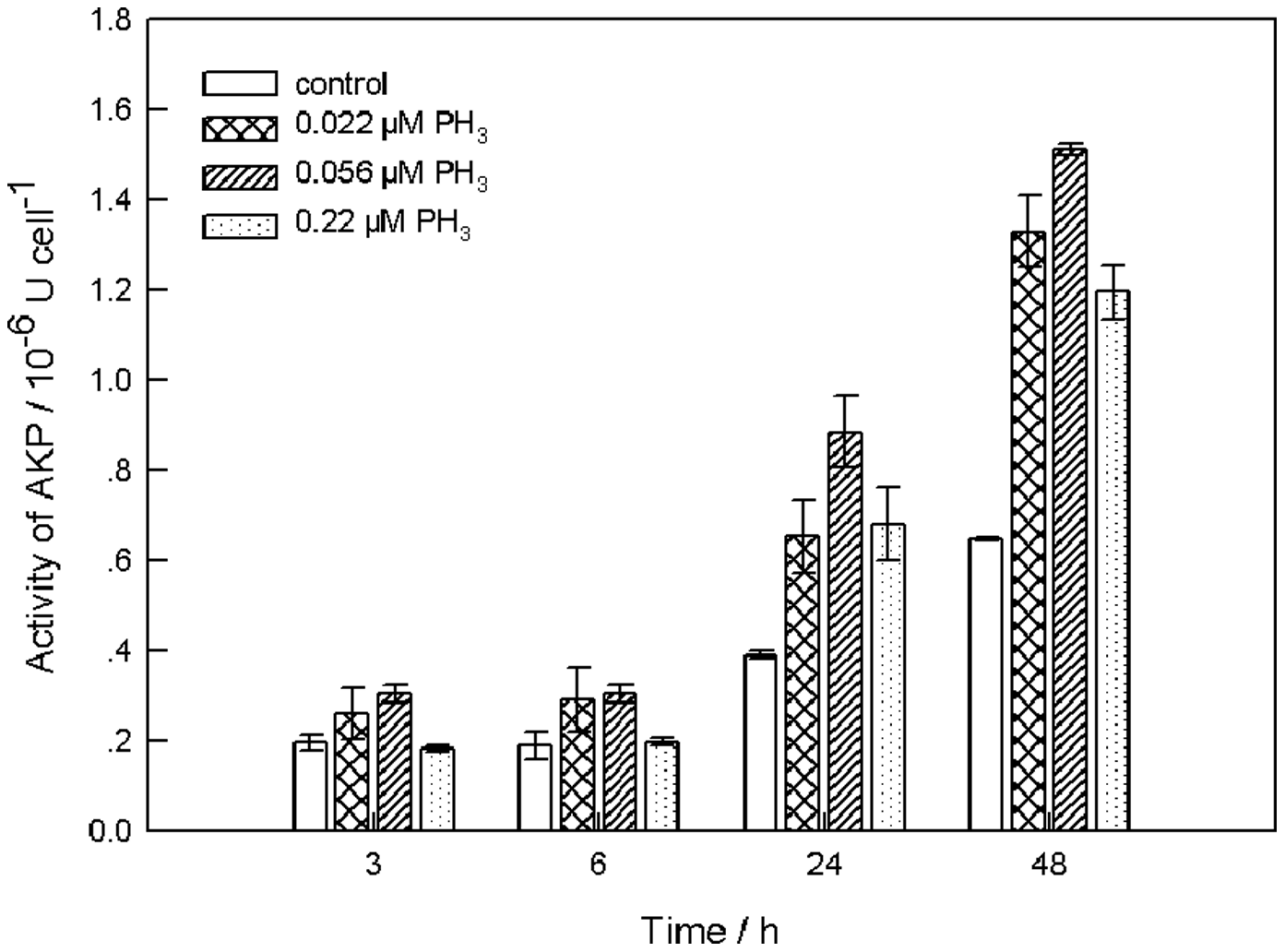
Effects of phosphine on AKP activity in *T*. *pseudonana*. Enzymatic activity, 1 U Cell^−1^ is expressed as 1µg P-nitrophenol liberated by AKP per algal cell for 1.5 h. The error bars represent standard deviations from three replicate treatments.

Results of the previous experiment revealed that *Tp*PHO gene expression varied with phosphine concentration; in other words, phosphine may affect the P metabolism of phosphate. Phosphine concentration markedly affected gene expression on the first day of the experiment. To further confirm that phosphine affects P metabolism, a short-term experiment to measure the effect of phosphine on AKP activity was conducted with different phosphine concentrations and with the influence of growth stage eliminated. Figure 4 shows the results of the experiment. AKP activity increased with culture age in all treatments, and it was generally higher in the three phosphine treatments than in the control. The highest level, 1.511×10^−6^ U cell^−1^, was observed in the treatment with the middle concentration of phosphine (0.056 µM PH_3_) after 48 h. The variation in AKP activity did not completely coincide with the variation in the *Tp*PHO transcriptional level. However, low phosphine concentration (0.022 µM PH_3_) had a greater effect (i.e., promotion) on both parameters than high concentration (0.22 µM PH_3_).

## Discussion

In this study, effects of phosphine on the cell density and specific growth rates in *T*. *pseudonana* were investigated. The phosphine treatments had higher specific growth rates during the early culture period; however, with continuous introduction of phosphine and prolonged stress time, specific growth rates of the phosphine treatments became lower than that of controls. During the decline phase, the mortality rate of the high-PH_3_ treatment was higher than that of the low-PH_3_ treatment; and the mortality rate of the low-PH_3_ treatment was higher than that of the control. These results suggest that phosphine has a dual effect on algae: phosphine can stimulate cells to reproduce at a low concentration, while has inhibitory effect on cell vitality at a high concentration. Niu et al. [20] reported a similar result for *Microcysis aeruginosa*. Before the microalgae enter a low growth phase and even die, a transient higher growth rate of phosphine treatment during the first few days may reflect a kind of "stress response" of the organisms to phosphine. Chefurka et al. [18] found that phosphine can affect the electron transport chain, and Nocter and Foyer [34] suggested that disturbances of the photosynthetic electron transport chain can result in oxidative stress in plants. Phosphine could influence the anti-oxidative defense system. Bolter and Chefurka [17] reported that phosphine induced an increase of superoxide dismutase activity, and the anti-oxidation mechanisms could protect the cell against oxygen-derived radicals. Low phosphine concentrations may induce the production of reactive oxygen species (ROS), which could stimulate anti-oxidative mechanisms. The activation of the anti-oxidative system could help enhance the cell’s tolerance to radicals, meaning that phosphine at low concentrations is beneficial to cell growth. In contrast, high concentrations of phosphine would induce too much ROS for the anti-oxidative system, resulting in lipid peroxidation and reduce algal growth subsequently [17], [35]. The effect of phosphine is likely consistent with the effect of ROS. Pelicano et al. [36] demonstrated that ROS have a dual effect on cell growth: low levels of ROS induced cell proliferation, whereas excess ROS led to cell death.

The result of the phosphine experiments clearly indicated that *Tp*PHO expression was markedly promoted by phosphine in the phosphate-deficient medium. On the other hand, high phosphine concentration had inhibitory effects on *Tp*PHO transcription during the decline phase (Figure 3A). Comparison of the phosphine treatment with the phosphate treatment of the same concentration revealed that the *Tp*PHO transcriptional level of the former was much higher than that of the latter (Figure 3B and 3C). Therefore, phosphine appears to influence the algae in a manner other than by being oxidized to phosphate. A similar result was obtained in the 4 µM phosphate culture: *Tp*PHO exhibited much higher expression in the phosphine treatments than in the control (Figure 3D). Previous studies have found a strong relationship between phosphate uptake and the expression of phosphate transporter genes [21]. For example, the expression of PHO84 and PHO89 in *Saccharomyces cerevisiae* was controlled by the phosphate concentration [37]. In *Tetraselmis chui*, *Tc*PHO expression responded to the external concentration of phosphate [25]. Thus, under the same concentration of phosphate and the same experimental conditions, the observed effects of phosphine on *Tp*PHO transcriptional level means that phosphine influenced phosphate uptake. These results suggest that phosphine triggers a P-deficiency signal to stimulate transcription of phosphate transporter genes, but the origin and nature of this signal is unknown. It could be considered a survival strategy to overcome the pressure from phosphine. Growing evidence supports the role of ROS as secondary messengers in signal pathways to regulate cell division [36], [38], [39]. This might be a mechanism that can explain the effect of phosphine on gene expression.

The response of *Tp*PHO transcription to phosphine increased with the concentration of phosphate in culture (Figure 3E). The highest level of *Tp*PHO transcription was observed in the low-PH_3_ treatment in the phosphate-4 µM culture(4 µM PO_4_^3−^ +0.022 µM PH_3_), which means that phosphine gave a bigger boost to *Tp*PHO transcription as the phosphate increased in the culture. *Tp*PHO expression is linked to the level of ambient phosphate. Nutritional conditions can impact the physiological characteristics of plants, such as signal transduction, enzymatic activity, and gene expression [40]. Algal cell vitality was strengthened at higher phosphate concentrations, thereby making proteins efficient in transmitting or responding to signals. Consequently, with higher phosphate concentration, the *Tp*PHO transcriptional level is increased and the capacity for phosphate transport is improved subsequently.

As the culture aged, the sum of the added concentration of phosphine in the culture increased to a high level. Hsu et al. [35] reported that phosphine can induce DNA damage in rats. Meanwhile, algal cell vitality weakened in the later stage of the growth cycle. Therefore, gene expression was inhibited by phosphine. The observation that a low phosphine concentration boosted *Tp*PHO gene expression was consistent with the variation of growth rate. Moreover, the effect of phosphine on phosphate uptake would further influence algal growth. This deduction was in agreement with published studies about the effect of phosphate on algal growth [20]. Furthermore, the response of *Tp*PHO expression was more sensitive than that of algal growth.

The data revealed that changes of AKP activity and the *Tp*PHO transcriptional level caused by phosphine were similar. Values of the two parameters in the phosphine treatments were higher than those in the control. A considerable amount of research has shown that AKP activity is negatively related to phosphate concentration [41], [42]. In the present study, if all of the phosphine was oxidized to phosphate, the concentration of phosphate in the medium should have increased as the added concentration of phosphine increased. Thus, AKP activity should have decreased with the increasing concentration of phosphine. In the experiment, AKP activity increased with the increase of phosphine in the range of 0 to 0.056 µM. This increasing AKP activity suggests that phosphine has other ways of influencing algae in addition to being oxidized to phosphate for uptake. These results demonstrate that phosphine influences the P metabolism of phosphate. The toxicity of phosphine is the reason why AKP activity in the high phosphine treatment was lower than that in the low phosphine treatment.

AKP hydrolyzes a variety of forms of organic P and releases P, making it available for uptake [26]. Meanwhile, the phosphate transporter is supposed to be the first step in phosphate transport [25]. However, there is not a direct relationship between AKP and the phosphate transporter. The only connection is that they are closely related to P metabolism and improved by phosphate deficiency. The similar variations in these parameters in response to varying levels of phosphine indicate that phosphine triggers a P-deficiency signal.

### Conclusions and Perspectives

Our experimental data demonstrate that phosphine influences algae in a way other than by oxidation to release P for uptake. Both *Tp*PHO transcriptional level and AKP activity of *T. pseudonana* increased at low phosphine concentration. These response mechanisms suggest that phosphine improves phosphate uptake and utilization, probably by triggering a P-deficiency signal. On the other hand, the toxicity of phosphine can induce damage to DNA and the plasma membrane system, thus gene expression and enzyme activity were inhibited at high phosphine concentrations. These variations indicate the dual effect of phosphine on *T. pseudonana*. However, different phytoplankton groups have different ecophysiological strategies and molecular level response mechanisms, resulting in different responses to phosphine. Whether other phytoplankton species have the same response to phosphine remains to be determined. In addition, a better understanding of the effect of phosphine on phosphorus metabolism will help explain the mechanism by which phosphine influences phytoplankton. Studies of the effect of phosphine on the electron transport chain and anti-oxidative system will be helpful to explain this mechanism.
